# Supplementary material for: Autonomous and non-cell autonomous role of cilia in structural birth defects in mice
Source: PLoS Biol. 2023 Dec 11;21(12):e3002425. doi: 10.1371/journal.pbio.3002425 (PMC10735189; doi:10.1371/journal.pbio.3002425)
Supplement: S1 Table — *Subset of -/- embryos that died (no heartbeat or blood flow, or already undergoing resorption). (DOCX) [file pbio.3002425.s001.docx]

|  | +/+ | +/- | -/- | -/-* |
| --- | --- | --- | --- | --- |
| E9 | 15 | 30 | 23 | 0 |
| E10 | 18 | 16 | 20 | 0 |
| E11 | 7 | 20 | 10 | 2 |
| E12 | 16 | 26 | 15 | 5 |
| E13 | 19 | 54 | 15 | 10 |
| E14 | 17 | 30 | 5 | 2 |
| E15 | 9 | 14 | 2 | 1 |
| E16 | 5 | 2 | 0 | 0 |

**S1 Table. *Midgestation lethality of Ift140^null1/null1^ embryo***

*Subset of -/- embryos that died (no heartbeat or blood flow, or already undergoing resorption.
