# Supplementary material for: Autonomous and non-cell autonomous role of cilia in structural birth defects in mice
Source: PLoS Biol. 2023 Dec 11;21(12):e3002425. doi: 10.1371/journal.pbio.3002425 (PMC10735189; doi:10.1371/journal.pbio.3002425)
Supplement: S2 Table — †Mouse Genome Informatics (MGI) https://www.informatics.jax.org/. (DOCX) [file pbio.3002425.s002.docx]

| Cre | Promotor | Tamoxifen-Driven? | Cre localization | Embryonic day expressed | Jax MGI Symbol^†^ | Citation |
| --- | --- | --- | --- | --- | --- | --- |
| *CAGGCre-ER* | *ACTB*, actin, beta (chicken) | YES | Consistently Expressed | Oocyte onwards | Tg(CAG-cre/Esr1*)5Amc | [1] |
| *Wnt1-Cre* | *Wnt1,* wingless-related MMTV integration site 1 (mouse) | NO | Midbrain, neural crest and derivatives | E8 | H2az2^Tg(Wnt1-cre)11Rth^ | [2, 3] |
| *Tbx18-Cre* | *T-box18,* T-box transcription factor 18 (mouse) | NO | Mesenchymal derivatives, epicardial progenitors | E9.5 | Tg(Tbx18-icre)3Fech | [4] |
| *Foxa2Cre-ER* | *Foxa2,* forkhead box A2 (mouse) | YES | Node, notochord, floorplate, and endoderm | E7.5 | Foxa2^tm2.1(cre/Esr1*)Moon^ | [5] |
| *Mef2c-Cre* | *Mef2c,* myocyte enhancer factor 2C (mouse) | NO | Developing myotome, anterior heart field (right ventricle, outflow tract, and ventricular septum) | E7.5 | Tg(Mef2c-cre)1Blk | [6, 7] |
| *Tie2-Cre* | *Tie2,* receptor tyrosine kinase Tek (mouse) | NO | Endothelial cells | E7.5 | Tg(Tek-cre)12Flv | [8] |

**S2 Table. Summary of Cre lines used in this study.**  ^†^Mouse Genome Informatics (MGI) https://www.informatics.jax.org/

**References**

1. Hayashi S, McMahon AP. Efficient recombination in diverse tissues by a tamoxifen-inducible form of Cre: a tool for temporally regulated gene activation/inactivation in the mouse. Dev Biol. 2002;244(2):305-18. Epub 2002/04/12. doi: 10.1006/dbio.2002.0597. PubMed PMID: 11944939.

2. Rowitch DH, B SJ, Lee SM, Flax JD, Snyder EY, McMahon AP. Sonic hedgehog regulates proliferation and inhibits differentiation of CNS precursor cells. J Neurosci. 1999;19(20):8954-65. Epub 1999/10/12. doi: 10.1523/JNEUROSCI.19-20-08954.1999. PubMed PMID: 10516314; PubMed Central PMCID: PMCPMC6782773.

3. Danielian PS, Muccino D, Rowitch DH, Michael SK, McMahon AP. Modification of gene activity in mouse embryos in utero by a tamoxifen-inducible form of Cre recombinase. Curr Biol. 1998;8(24):1323-6. Epub 1998/12/09. doi: 10.1016/s0960-9822(07)00562-3. PubMed PMID: 9843687.

4. Wang Y, Tripathi P, Guo Q, Coussens M, Ma L, Chen F. Cre/lox recombination in the lower urinary tract. Genesis. 2009;47(6):409-13. Epub 2009/05/06. doi: 10.1002/dvg.20515. PubMed PMID: 19415630; PubMed Central PMCID: PMCPMC2848076.

5. Park EJ, Sun X, Nichol P, Saijoh Y, Martin JF, Moon AM. System for tamoxifen-inducible expression of cre-recombinase from the Foxa2 locus in mice. Dev Dyn. 2008;237(2):447-53. Epub 2007/12/28. doi: 10.1002/dvdy.21415. PubMed PMID: 18161057.

6. Verzi MP, McCulley DJ, De Val S, Dodou E, Black BL. The right ventricle, outflow tract, and ventricular septum comprise a restricted expression domain within the secondary/anterior heart field. Dev Biol. 2005;287(1):134-45. Epub 2005/09/29. doi: 10.1016/j.ydbio.2005.08.041. PubMed PMID: 16188249.

7. Heidt AB, Black BL. Transgenic mice that express Cre recombinase under control of a skeletal muscle-specific promoter from mef2c. Genesis. 2005;42(1):28-32. Epub 2005/04/14. doi: 10.1002/gene.20123. PubMed PMID: 15828002.

8. Koni PA, Joshi SK, Temann UA, Olson D, Burkly L, Flavell RA. Conditional vascular cell adhesion molecule 1 deletion in mice: impaired lymphocyte migration to bone marrow. J Exp Med. 2001;193(6):741-54. Epub 2001/03/21. doi: 10.1084/jem.193.6.741. PubMed PMID: 11257140; PubMed Central PMCID: PMCPMC2193418.
