# Supplementary material for: Autonomous and non-cell autonomous role of cilia in structural birth defects in mice
Source: PLoS Biol. 2023 Dec 11;21(12):e3002425. doi: 10.1371/journal.pbio.3002425 (PMC10735189; doi:10.1371/journal.pbio.3002425)
Supplement: S3 Table — †When using tamoxifen-inducible Cre (CAGGCre-ER, Foxa2Cre-ER), tamoxifen was administered to pregnant mothers at embryonic ages listed. (DOCX) [file pbio.3002425.s003.docx]

| **Parent Genotypes** | | | **Tamoxifen Used^†^** | **Embryo Genotypes** | |
| --- | --- | --- | --- | --- | --- |
| Father |  | Mother | (Embryonic Age Given) | Control embryos | Experimental embryos |
| *Ift140^+/null1^* | x | *Ift140^+/null1^* | No | *Ift140^+/+^* | *Ift140^null1/null1^* |
| *Ift140^+/220^* | x | *Ift140^+/220^* | No | *Ift140^+/+^* | *Ift140^220/220^* |
| *Ift140^+/null1^ + Wnt1-Cre* | x | *Ift140^flox/null1^* | No | *Ift140^flox/+^, Wnt1-Cre^+^* | *Ift140^flox/null1^, Wnt1-Cre^+^* |
| *Ift140^+/null1^ + Tbx18-Cre* | x | *Ift140^flox/null1^* | No | *Ift140^flox/+^, Tbx18-Cre^+^* | *Ift140^flox/null1^, Tbx18-Cre^+^* |
| *Ift140^+/null1^ + Tie2-Cre* | x | *Ift140^flox/null1^* | No | *Ift140^flox/+^, Tie2-Cre^+^* | *Ift140^flox/null1^, Tie2-Cre^+^* |
| *Ift140^+/null1^ + Mef2c-Cre* | x | *Ift140^flox/null1^* | No | *Ift140^flox/+^, Mef2c-Cre^+^* | *Ift140^flox/null1^, Mef2c-Cre^+^* |
| *Ift140^+/null1^ + CAGGCre-ER* | x | *Ift140^flox/null1^* | Yes (E5.5) | *Ift140^flox/+^, CAGGCRE-ER^+^* | *Ift140^flox/null1^, CAGGCRE-ER^+^* |
| *Ift140^+/null1^ + CAGGCre-ER* | x | *Ift140^flox/null1^* | Yes (E7.5 or E8.5) | *Ift140^flox/+^, CAGGCRE-ER^+^* | *Ift140^flox/null1^, CAGGCRE-ER^+^* |
| *Ift140^+/null1^ + Foxa2Cre-ER* | x | *Ift140^flox/null1^* | Yes (E6.5, E7.5, or E8.5) | *Ift140^flox/+^, Foxa2Cre-ER^+^* | *Ift140^flox/null1^, Foxa2Cre-ER^+^* |

**S3 Table. Summary of mouse breeding used to generate embryos analysed in this study**

^†^ When using tamoxifen-inducible Cre (*CAGGCre-ER, Foxa2Cre-ER*), tamoxifen was administered to pregnant mothers at embryonic ages listed.
