# Supplementary figures and images for: Autonomous and non-cell autonomous role of cilia in structural birth defects in mice
Source: PLoS Biol. 2023 Dec 11;21(12):e3002425. doi: 10.1371/journal.pbio.3002425 (PMC10735189; doi:10.1371/journal.pbio.3002425)

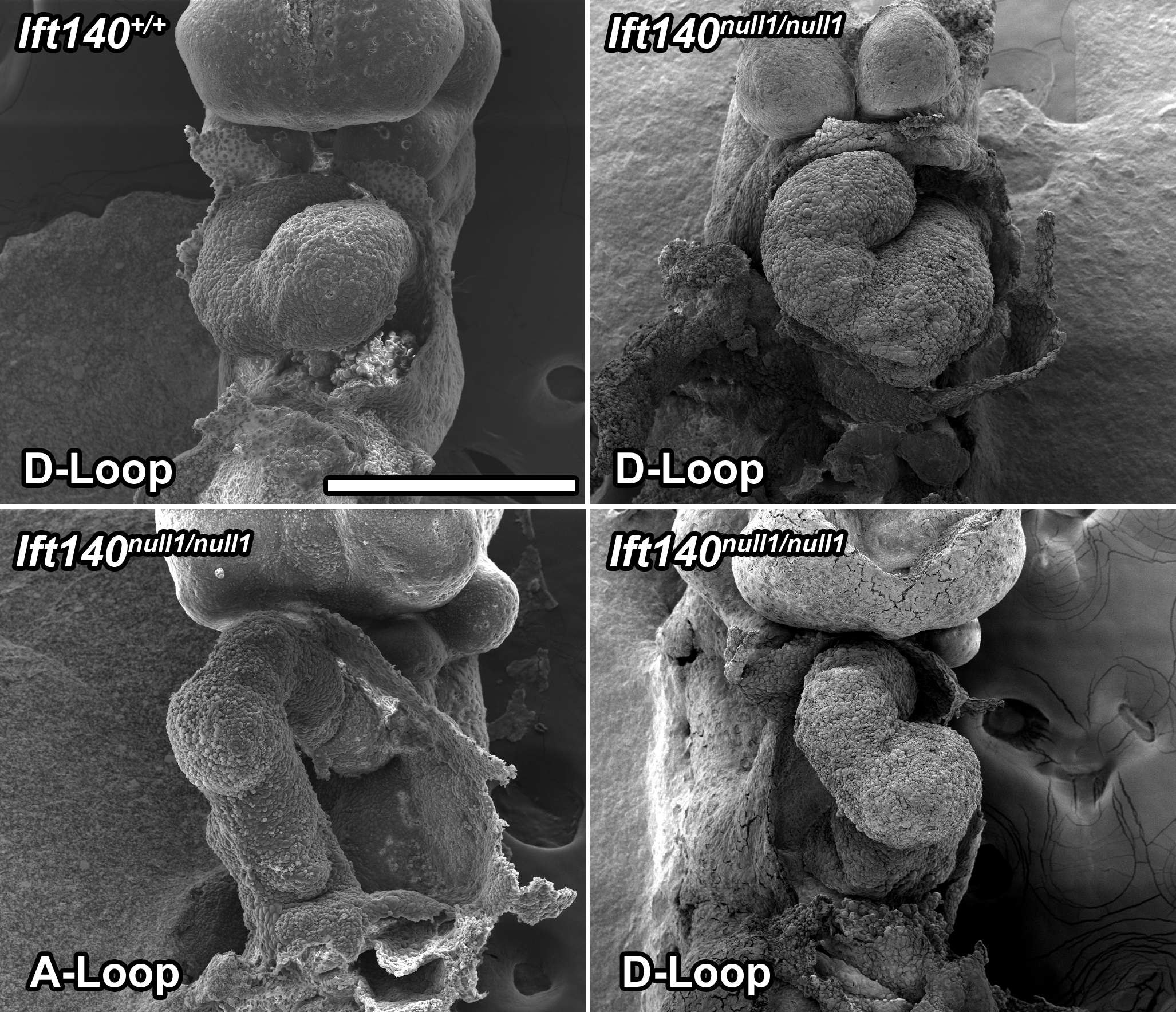

Supplement: S1 Fig — Scanning electron microscopy reveals heart looping defects in Ift140null1/null1 embryos. Scale bar is 0.5 mm. (TIF) [file pbio.3002425.s004.tif]

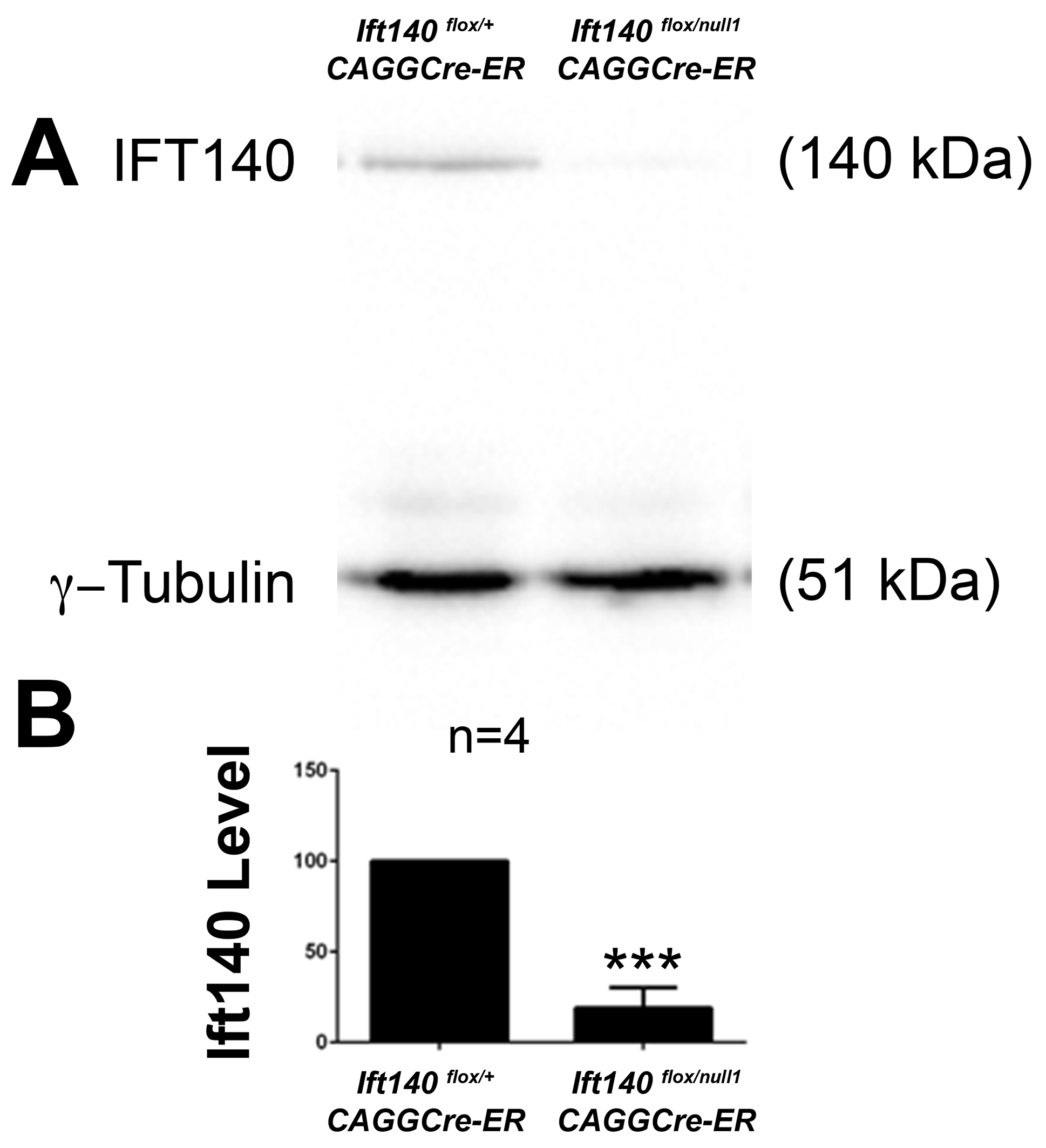

Supplement: S2 Fig — (A) Western blot showing IFT140 levels in whole embryo lysates (Ift140flox/+ CAGGCre-ER+ vs. Ift140flox/null1 CAGGCre-ER+) 48 h after treatment of the mother with 0.1 ml (1 mg) tamoxifen administered by oral gavage. Embryos were treated at E9 and harvested at E11. γ-tubulin is a loading control. (B) Quantification of the extent of IFT140 reduction 48 h after treatment of the mother with tamoxifen. Level of IFT140 was normalized between embryos using γ-tubulin and then experimental and control embryos within a litter were ratioed with controls set to 100%. Raw counts were normalized to controls from the same litter. ***p < 0.0001, unpaired Student t test. Error bar is standard deviation. The data underlying this figure can be found in Supporting information S1 Data. (TIF) [file pbio.3002425.s005.tif]

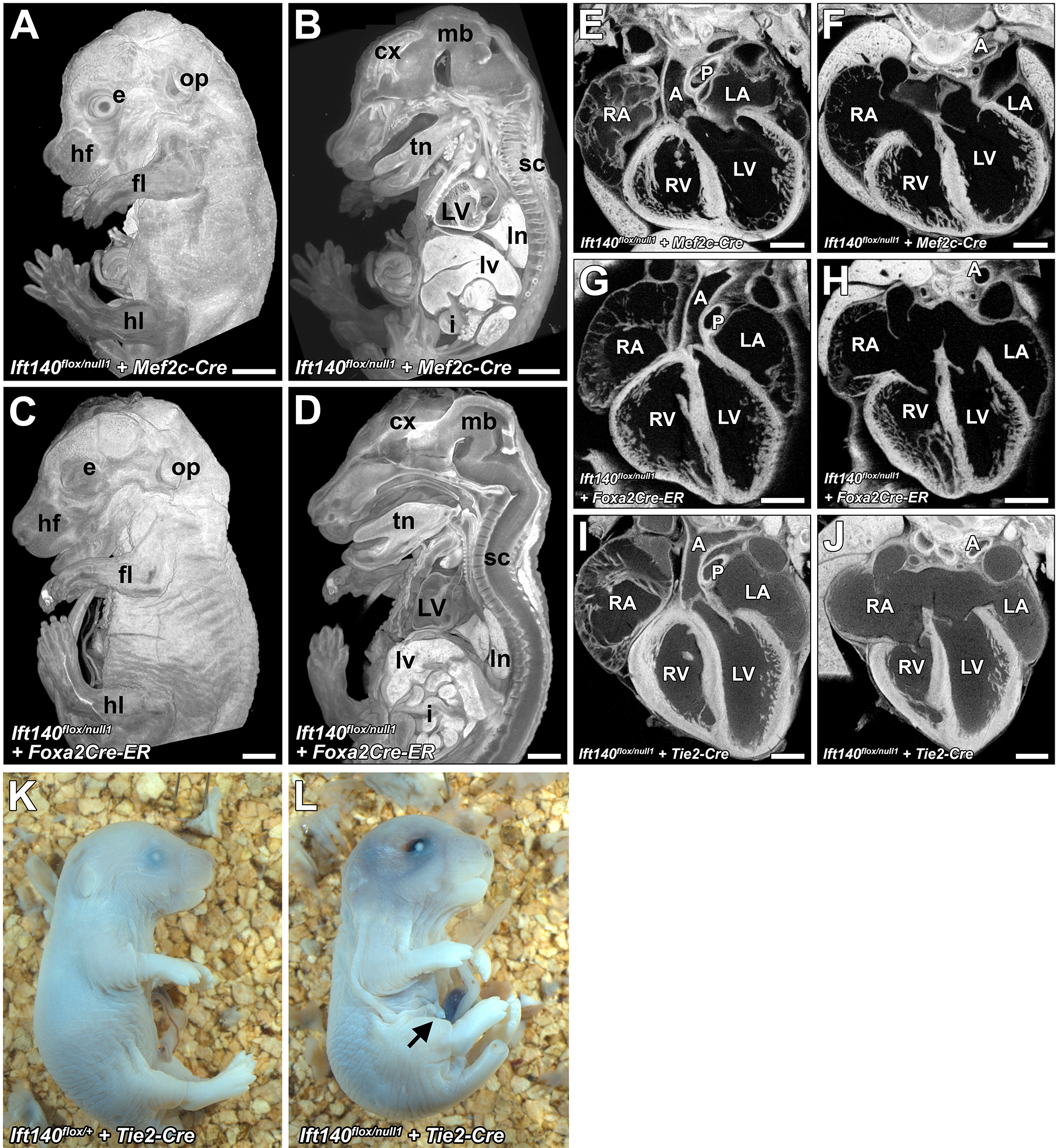

Supplement: S3 Fig — (A–D) Deletion of Ift140 by Mef2c-Cre or by tamoxifen-induced Foxa2Cre-ER (tamoxifen administered at E6.5, E7.5, or E8.5) display normal whole body gross anatomy. (E–J) Deletion of Ift140 by Mef2c-Cre, Tie2-Cre, or by tamoxifen-driven Foxa2Cre-ER did not affect cardiac and great vessel anatomy. (K, L) Deletion of Ift140 by Tie2-Cre results in eyelid closure defects and supernumerary mammary glands (arrow). LV: left ventricle; cx: cerebral cortex; sc: spinal cord; mb: midbrain; fl: forelimb; hl: hindlimb; e: eye; op: otic placode; hf: hair follicles; lv: liver; ln: lungs; i: small intestine; A: aorta; P: pulmonary trunk; LV: left ventricle; RV: right ventricle; LA: left atria; RA: right atria. Scales bars: (A–D) = 1 mm, (E–J) = 0. 5 mm. (TIF) [file pbio.3002425.s006.tif]

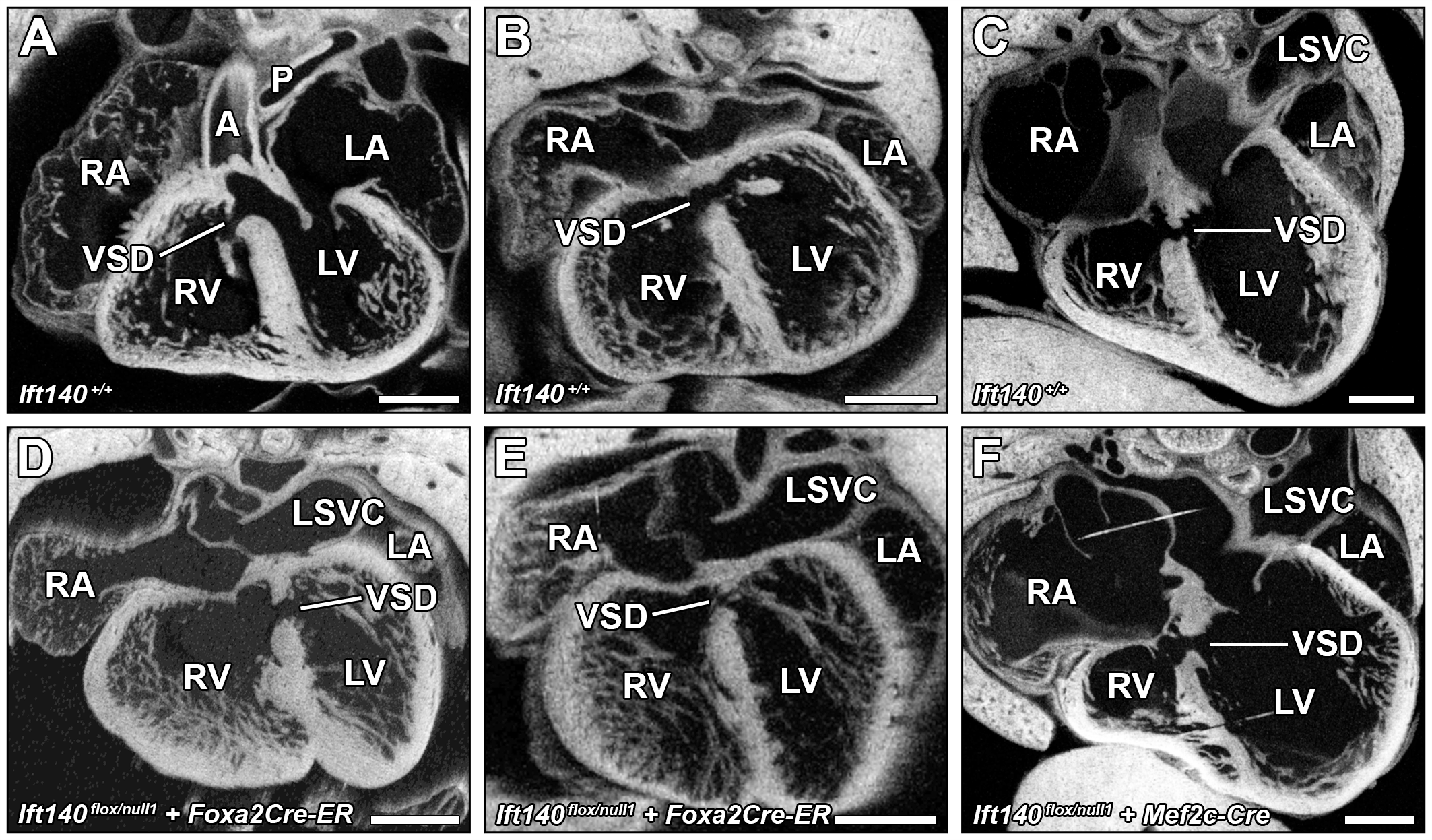

Supplement: S4 Fig — (A–C) A small number of littermate controls (Ift140+/+) collected from tamoxifen treated litters were found to have small VSDs. (D–F) Similar small ventricular septal defects were also seen in a small number of embryos with tamoxifen-driven Cre-specific knockdown, including Foxa2Cre-ER (D, E) and Mef2c-Cre (F). As these defects were seen across both wild type and experimental knockdown groups, they were excluded from phenotypic analysis and categorized as possible experimental artifacts. A: aorta; P: pulmonary trunk; LV: left ventricle; RV: right ventricle; LA: left atria; RA: right atria; VSD: ventricular septal defect; LSVC: left superior vena cava. All scales bars = 0.5 mm. (TIF) [file pbio.3002425.s007.tif]

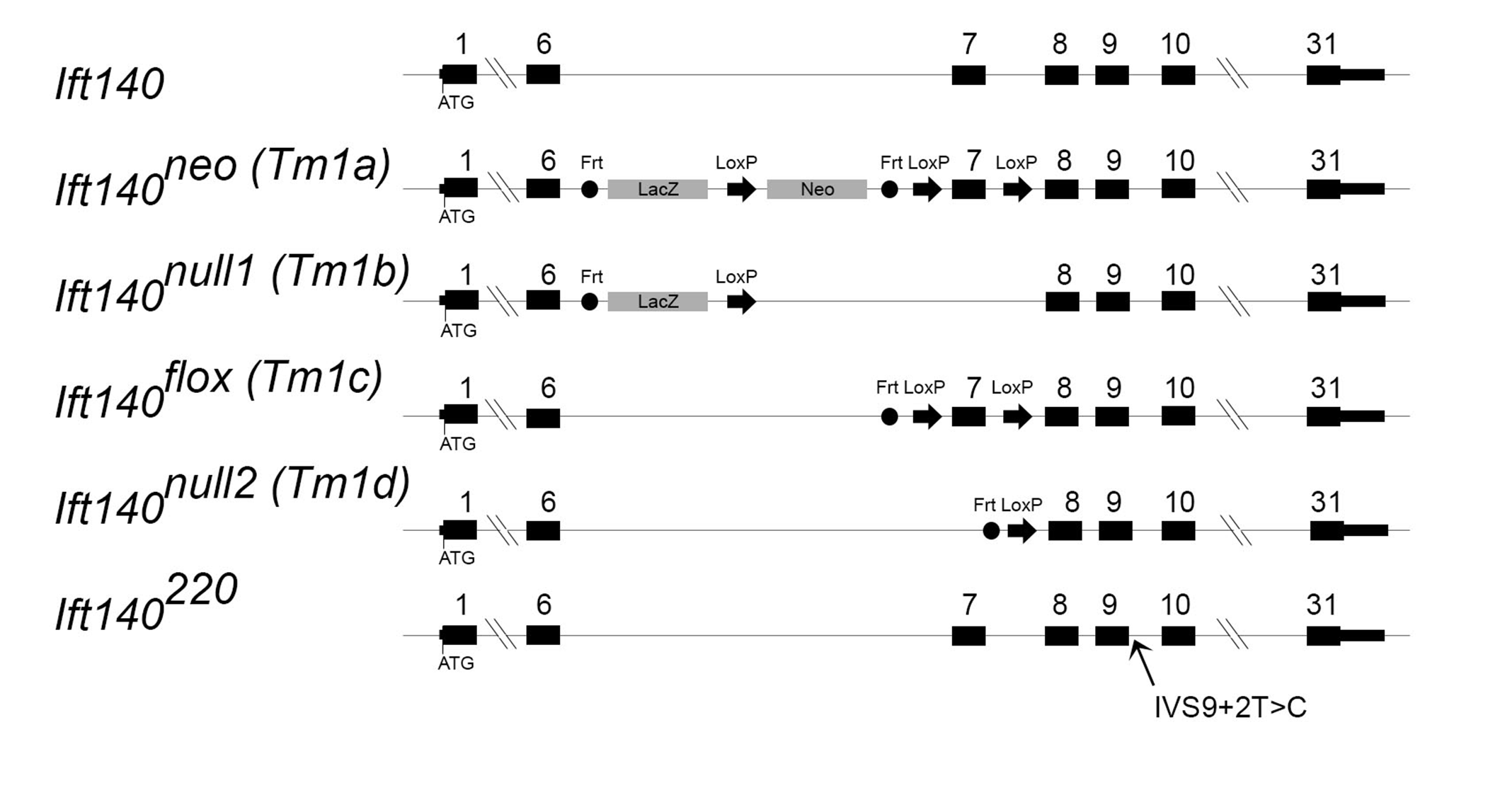

Supplement: S5 Fig — Numbered black boxes indicate exon protein-coding regions; arrows: LoxP sites; ATG: start codon, Frt: flippase recombination target recognition site; LacZ: beta-galactosidase gene; Neo: neomycin-resistance gene. (TIF) [file pbio.3002425.s008.tif]

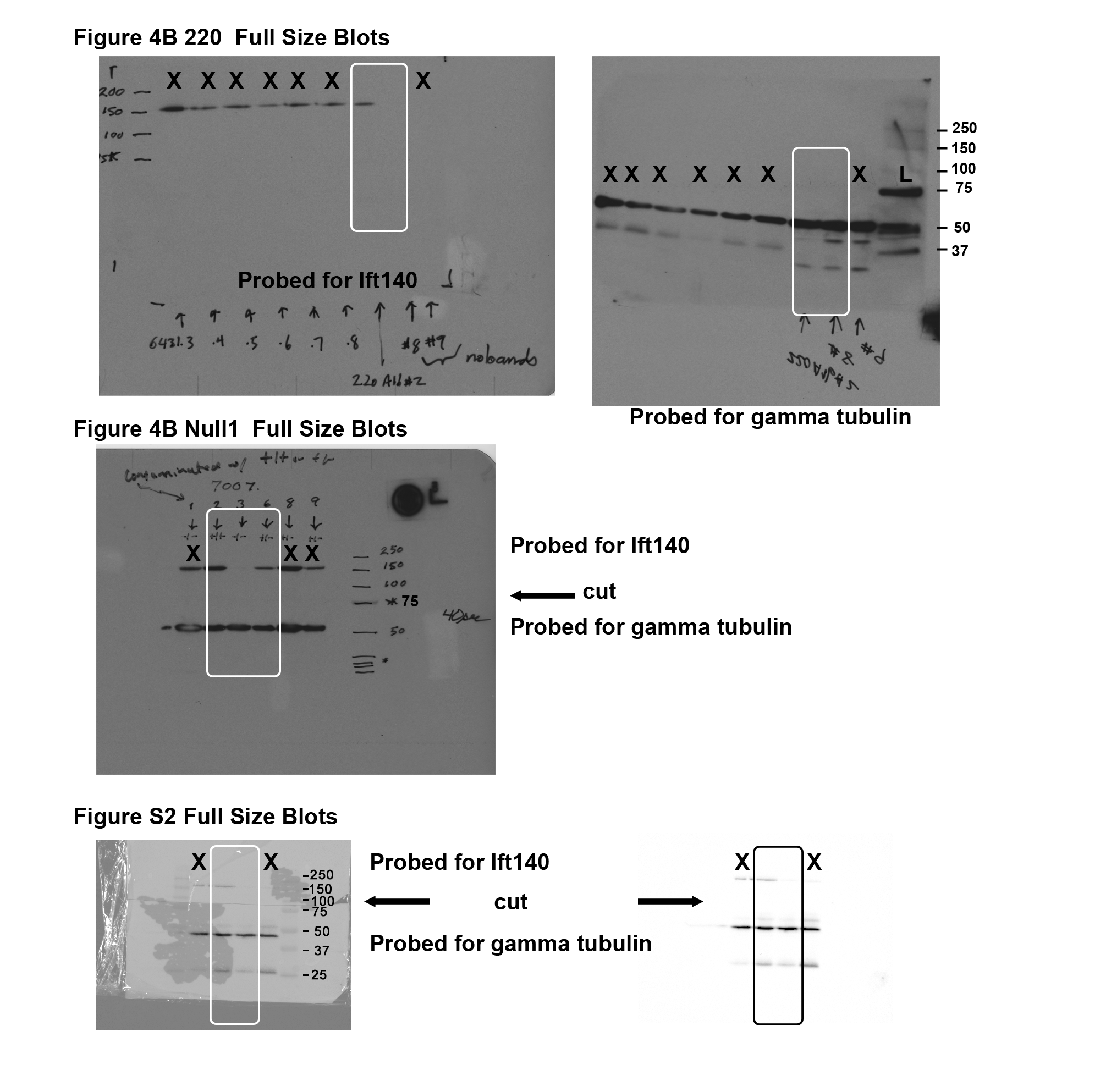

Supplement: S1 Raw Images — Top blots: Left membrane was probed for Ift140 while the right was probed for gamma tubulin. The lanes used in Fig 4B are in the white box. Middle blot: Membrane was cut (arrow) and the top half probed for Ift140 and the lower half probed for gamma tubulin. The lanes used in Fig 4B are in the white box. Bottom blot: Membrane was cut (arrow) and the top half probed for Ift140 and the lower half probed for gamma tubulin. The left image is the western blot superimposed on an image of the membrane while the right image is only the western blot. The lanes used in S2 Fig are in the boxes. (TIF) [file pbio.3002425.s009.tif]
