# Supplementary material for: Autonomous and non-cell autonomous role of cilia in structural birth defects in mice
Source: PLoS Biol. 2023 Dec 11;21(12):e3002425. doi: 10.1371/journal.pbio.3002425 (PMC10735189; doi:10.1371/journal.pbio.3002425)
Supplement: Attachment — Submitted filename: Response to editorial requests.docx [file pbio.3002425.s020.docx]

Response to editorial requests:

1) TITLE: Given the public interest in this topic, we think the title of your piece should be edited to indicate that the study was done in mice. We therefore suggest it be changed to something like:

"Autonomous and non-cell autonomous role of cilia in structural birth defects in mice"

Title changed as suggested.

2) ETHICS STATEMENT: Please update the ethics statement, in your materials and methods section, to include the specific national or international regulations/guidelines to which your animal care and use protocol adhered. Please note that institutional or accreditation organization guidelines (such as AAALAC) do not meet this requirement.

Methods section updated with below ethics statement:

"Mouse research was carried out at the University of Massachusetts Chan Medical School with IACUC approval (PROTO201900265) and at the University of Pittsburgh with IACUC approval (Protocol 21120410). These IACUCs follow the regulations of US Department of Agriculture Animal Welfare Act and the standards/principles of the Public Health Service Policy on Humane Care and Use of Laboratory Animals, AVMA Guidelines on Euthanasia, U.S. Government Principles for the Utilization and Care of Vertebrate Animals Used in Testing, Research and Training, and the Guide for the Care and Use of Laboratory Animals."

3) DATA: Thank you for providing the data underlying your figures as a supplemental file. Can you please update this to add the data from the supplemental figures as well (ex the underlying data for Supplemental Figure 2B)? Please also add a note to each relevant figure legend that directs readers to this file. You can add the sentence, "the data underlying this figure can be found in supplemental file RawDataForFigures.

Full size western blot for Figure 2 can be found in "S1_Raw_Images" (bottom left image).

Raw counts were normalized to controls from the same litter: control n=4 ( 100, 100, 100, 100) and experimental n=4 (9.18, 12.0, 21.1, 33.9). Figure S2 legend updated to include information.

Figure legends also updated as directed.

4) BLOTS: Thanks also for providing the uncropped western blots related to this paper as a supplemental figure. A few requests regarding this data:

a.  Rather than presenting these as a figure, can you provide them as a supplemental file, titled "S1_Raw_Images"?

Figure S5 removed from text. Blots renamed to "S1_Raw_Images" as suggested

b. We ask that you please add a few more annotations to this file. Specifically, please indicate molecular weight markers. For bands that were not presented in the main figure, please add an 'x' over each irrelevant well (without obscuring the image).

Changes made to "S1_Raw_Images" as suggested

c. The blots provided for Fig 4B, Gamma tubulin seems to be slightly cropped. Please provide a less cropped version of this figure. If it is difficult to see the full membrane, please just provide the full page image generated by the scanner.

The contrast was adjusted slightly on 4B gamma tubulin gel in "S1_Raw_Images" to show that this is the whole blot.

When putting the molecular markers on the gamma tubulin blot for 4B 220, it was found that the blot was upside down. The lanes are correct, it is only that the bands were upside down.

This blot has been flipped so it is now correct.

The gamma tubulin bands in 4B 220 have also been corrected

5) CODE: Per journal policy, if any code was generated to support the conclusions of your manuscript, we would require that you make it available without restrictions upon publication. Please ensure that any code is sufficiently well documented and reusable, and that your Data Statement in the Editorial Manager submission system accurately describes where your code can be found.

No code was written

As you address these items, please take this last chance to review your reference list to ensure that it is complete and correct. If you have cited papers that have been retracted, please include the rationale for doing so in the manuscript text, or remove these references and replace them with relevant current references. Any changes to the reference list should be mentioned in the cover letter that accompanies your revised manuscript.

We expect to receive your revised manuscript within two weeks.
