# Supplementary material for: Autonomous and non-cell autonomous role of cilia in structural birth defects in mice
Source: PLoS Biol. 2023 Dec 11;21(12):e3002425. doi: 10.1371/journal.pbio.3002425 (PMC10735189; doi:10.1371/journal.pbio.3002425)
Supplement: Attachment — Submitted filename: Response to reviewer comments.docx [file pbio.3002425.s019.docx]

**Response to reviewer comments for manuscript PBIOLOGY-D-23-01584R1: Autonomous and non-cell autonomous etiology of ciliopathy associated structural birth defects**

The following changes have been made in response to the reviewers’ comments.

**Reviewer #1:**

*1) "From previous studies with various cilia-less mutant mice such as Kif3a mutant, the first stage during development that requires cilia is E7.5-8.0 when node cilia play a role in left-right (L-R) patterning. As expected, the Ift40 null mutant exhibit L-R defects. In conditional deletion with CAG-CreER, however, administration of Tmx at E7.5 failed to induce L-R defects.*

*This is most likely due to the time required for Cre-mediated deletion and to the stability of Ift40 protein. Thus, 19 % of Ift40 still remained 48 hours after Tmx treatment (Fig. S3). It was necessary to treat embryos with Tmx as early as E5.5, to see L-R defects. Tmx treatment at E6.5 resulted in L-R defects at a lower frequency. Are almost all node cilia lost in embryos treated at E5.5 and only a fewer cilia in those treated at E6.5? This can be easily examined by SEM."*

While we agree with reviewer #1 that physical visualization of nodal cilia by SEM in Tmx treated embryos would be intriguing, we politely disagree to its inclusion to this paper for the following reasons.

1. While conceptually, conducting SEM on node cilia is indeed easy. Practically, it is very difficult. E7.5 embryos are extremely small (only ~15k cells PMID: 19758561, some of which are needed for genotyping), and when prepared for SEM they become extremely fragile, making mounting for SEM very tricky with a high failure rate. Additionally, the node itself is comprised of less than 100 cells, and bowl shaped, making it difficult for achieving proper orientation to optimally visualize cilia.
2. We have already quantified the left-right phenotypic output, making visualization of nodal cilia somewhat superfluous
3. It may be hard to interpret any SEM data we do collect. e.g. We may see a huge knock down in the number of nodal cilia, but interpretation is problematic given the previous report that as few as two nodal cilia are all that is needed to break left-right signalling (PMID: 22233632)
4. Due to budgetary constraints, as we no longer carry these mouse lines in our mouse colonies we will not be able to pursue additional experiments.

*2) "The outflow tract (OFT) was severely affected in Ift40 null mutant and Tmx-treated conditional mutant embryos. On the other hand, The OFT remain nearly normal in Wnt1Cre conditional mutant. This was unexpected because the previous paper reported that Wnt1-Cre mediated deletion of Smo in neural crest cells result in OFT defects. Formally, the current data suggest that either Hedgehog signaling in neural crest cells was not downregulated by Wnt1Cre-mediated deletion or the OFT defects of Ift40 null mutant are not due to hedgehog deficiency in neural crest cells. Since this is an important finding of this paper, it can be clarified by examining the level of Shh level in neural crest cells with the Gli-LacZ."*

Actually the situation is more complex regarding the role of Shh in development of the heart. Development of the OFT requires Shh signalling not only in the neural crest (NC) cell lineage but also in the second heart field (SHF). Thus persistence truncus arteriosus (PTA), a phenotype seen in our *Ift140* mutants, is observed not only with conditional Shh deletion in NC cells (PMID: 17344228), but also with conditional Cre deletion of Shh/Smo in the second heart field (SHF) (PMID: 17344228; PMID: 18441277). However, we found conditional deletion in either lineage yielded no CHD phenotypes. Moreover, Cre deletion of Smo in the SHF via Mef2C-Cre (PMID: 17344228), the same Cre driver used in our study, has been shown to cause atrioventricular septal defects (AVSD), a phenotype also seen in our *Ift140* mutant, but this CHD phenotype also failed to emerge in the Mef2C-Cre deleted *Ift140* embryos.

Given no CHD phenotype was observed with Ift140 deletion using two Cre drivers known to yield PTA (Wnt1 and Mef2c-Cre) and PTA/AVSD (Mef2c-Cre) upon deletion of *Smo* or *Shh,* it would seem unlikely that our negative findings are due to failure of the Cre deletion to downregulate Shh signalling, but rather these CHD phenotypes (PTA/AVSD) may not arise from Shh deficiency. In support of this, we noted while disruption of Shh signalling is associated with shortening of the OFT, our Ift140 KO mice in fact showed the opposite, ie. significant lengthening (see below figure; now added as new panel “N” in Figure 3).

During heart development, SHF cells migrate into the heart tube to form the OFT. Shh signalling has been shown to suppress SHF cell differentiation into cardiomyocytes, thus allowing the SHF progenitors to expand in numbers, migrating to form and lengthen the OFT. With Shh deficiency, the SHF undergoes premature differentiation, thus causing OFT shortening, and failure in spiralling of the OFT. This is associated with disruption in neural crest migration into the OFT, with ensuing OFT septation and malalignment defects. The finding of OFT lengthening in the *Ift140* KO mice would suggest hedgehog signalling is preserved, and possibly even elevated. This would support our suggestion that the precise role of *Ift140* in hedgehog signalling may be context dependent. We have revised the discussion to include a brief consideration of the formal possibility of increased Shh signalling contributing to OFT defects in the Ift140 mutants. However, as development of the OFT is regulated by many cell signalling pathways in addition to Shh, including Fgf, Tgfb, Bmp, semaphorin, retinoic acid, and Pdgf (PMID: 33918884), we further discuss possible involvement of a non-cell autonomous role for cilia in the integration of multiple cell signaling pathways across different cell lineages to coordinate developmental processes regulating OFT morphogenesis. This has the added benefit that it can provide functional redundancy to protect the embryo from birth defects. We noted two other cell signalling pathways of specific relevance to this being Tgfb and Pdgf, as signal transduction for these two pathways are also known to be cilia transduced (PMID: 23264740; PMID: 23746451).

Note for completeness, we also incorporated into the Discussion, a brief consideration of the formal possibility that variability in Cre recombination levels could have caused incomplete knockdown of *Ift140*, thus resulting in variable or failure in knockdown of ciliation in the target tissues, and also subsequent failure to elicit CHD phenotypes. However, we point out that the CRE lines used in our study are well established and extensively utilized, and variable phenotypes from incomplete Cre deletion has not been reported previously. Moreover, similar analysis for other genes with these Cre drivers typically have yielded expected CHD phenotypes such as *Mef2C-Cre* deletion of *Lrp1* that fully replicated the DORV and AVSD phenotypes seen in the *Lrp1* KO mice (PMID: 32546759).

*3) "Non-cell autonomous cilia mediated interactions between multiple cell lineages" (page 18): this phrase can be discussed in more detail."*

Discussion text revised to address this point is shown below:

“While we found *Ift140* deficient MEFs showed loss of cilia and cilia transduced Shh signaling, no CHD phenotype was observed with *Ift140* deletion using either the *Wnt1* or *Mef2c-Cre* driver. This is despite previous studies showing PTA with *Smo* deletion by either *Wnt1* or *Mef2c-Cre* and AVSD with *Mef2c-Cre* [53, 54]. These findings would suggest the CHD phenotypes in the *Ift140* mutants are not likely to be the result of Shh deficiency. Consistent with this, we observed OFT lengthening in the *Ift140* mutants, while Smo or Shh deletion with *Mef2C-Cre* resulted in the opposite, the shortening of the OFT [53, 54]. Shh has been shown to promote OFT lengthening by suppressing SHF differentiation into cardiomyocytes and promoting SHF progenitor expansion, but with deficiency in Shh signaling, SHF progenitors are observed to undergo premature differentiation, resulting in OFT shortening. Thus the CHD phenotypes in the *Ift140* mutants may not reflect Shh deficiency, but rather disturbance of other cell signaling pathways known to regulate OFT development (Fgf, Tgfb, Bmp, semaphorin, retinoic acid, Pdgf; [55]). However, given the additional finding of ventralization of the neural tube, another formal possibility is an increase in hedgehog signaling arising from the *Ift140* deficiency. This is reminiscent of observations in other IFT mutants - *Ift122^sopb^* and *Ift139/Ttc21b^aln^* mutants [31, 32] and could account for OFT lengthening. Thus the precise role of *Ift140* in hedgehog signaling may be context dependent, with OFT development mediated by a non-cell autonomous role of cilia in the integration of multiple cilia transduced cell signaling pathways across different cell lineages. This could provide functional redundancy to prevent the emergence of lethal CHD phenotypes. We note in addition to Shh, Tgfβ and Pdgf, are two other cilia transduced cell signaling pathways with essential roles in OFT septation and alignment (PMID: 33918884),”

**Reviewer #2:**

*1. Could the authors please include the embryonic day at which each of the tissue-specific Cre's are expressed?*

New S2 Table added to manuscript to list all Cre lines used, including promotor, tissue expression, embryonic day expressed, MGI symbol, and citation.

*2. Related to this, Fig. 11A shows a timeline at which deletion of Ift140 results in various phenotypes. In Fig. 11B, could the phenotypes also be shown relative to the timeline e.g. as alluded to by the authors, heart looping defects would not be expected in Cre's that are expressed after E5.5.*

Figure 11 updated with a new panel added, Figure 11B to show timing of Tmx treatment and the resulting phenotypes (new Fig. 11B). Figure 11 panel C also updated to show embryonic stage when Cre expression occurs for each of the Cre line used (Fig. 11C).

*3. Fig 5E: Gli and Ptch1 expression are decreased, and Grem1 is increased. In the Cauli mouse mutant, Gli1, Ptch1 and Grem1 are increased leading to polydactyly. Could the authors please provide an explanation/rationale for the decreased Gli and Ptch1 in Ift140 mutants leading to polydactyly? Is the polydactyly preaxial e.g. duplication of digit 1?*

Discussion text expanded to highlight how complicated digit abnormalities are in mice with cilia defects. However, the Cauli mouse is not exactly as described by reviewer#2. *Gli1* was not examined in the *Cauli* mouse and *Ptch1* and *Grem1* were reduced posteriorly but expanded anteriorly (as outlined below). From PMID: 24009529 “Expression of *Ptch1*, the *Shh* receptor and a *Shh*-responsive gene, is reduced posteriorly but expanded anteriorly across limb buds of *Ift140^cauli/cauli^* mutants consistent with the presence of anterior Shh ([Figures 4E–H](https://aus01.safelinks.protection.outlook.com/?url=https%3A%2F%2Fjournals.plos.org%2Fplosgenetics%2Farticle%3Fid%3D10.1371%2Fjournal.pgen.1003746%23pgen-1003746-g004&data=05%7C01%7Crichard.francis%40jcu.edu.au%7C0e3550199a0945fb95fa08dbc989b5af%7C30a8c4e81ecd4f148099f73482a7adc0%7C0%7C0%7C638325365590237496%7CUnknown%7CTWFpbGZsb3d8eyJWIjoiMC4wLjAwMDAiLCJQIjoiV2luMzIiLCJBTiI6Ik1haWwiLCJXVCI6Mn0%3D%7C3000%7C%7C%7C&sdata=vJuj41vMtgehQHTPLGBG47e4T2d3LhX%2BYkxGPh89NMI%3D&reserved=0)). The distinct expression pattern of the Bmp antagonist *Grem1* seen in *Ift140^+/+^* limb buds is altered in *Ift140^cauli/cauli^* mutants ([Figures 4I–L, M′ and N′](https://aus01.safelinks.protection.outlook.com/?url=https%3A%2F%2Fjournals.plos.org%2Fplosgenetics%2Farticle%3Fid%3D10.1371%2Fjournal.pgen.1003746%23pgen-1003746-g004&data=05%7C01%7Crichard.francis%40jcu.edu.au%7C0e3550199a0945fb95fa08dbc989b5af%7C30a8c4e81ecd4f148099f73482a7adc0%7C0%7C0%7C638325365590237496%7CUnknown%7CTWFpbGZsb3d8eyJWIjoiMC4wLjAwMDAiLCJQIjoiV2luMzIiLCJBTiI6Ik1haWwiLCJXVCI6Mn0%3D%7C3000%7C%7C%7C&sdata=vJuj41vMtgehQHTPLGBG47e4T2d3LhX%2BYkxGPh89NMI%3D&reserved=0)). *Grem1* expression appears weaker in mutants compared to controls but is expanded both anteriorly and posteriorly. *Gli3*, a major mediator of *Shh* signalling in the limb, is spatially restricted in the anterior portion of the fore- and hindlimb buds of *Ift140^cauli/cauli^* embryos ([Figures 4M–P](https://aus01.safelinks.protection.outlook.com/?url=https%3A%2F%2Fjournals.plos.org%2Fplosgenetics%2Farticle%3Fid%3D10.1371%2Fjournal.pgen.1003746%23pgen-1003746-g004&data=05%7C01%7Crichard.francis%40jcu.edu.au%7C0e3550199a0945fb95fa08dbc989b5af%7C30a8c4e81ecd4f148099f73482a7adc0%7C0%7C0%7C638325365590237496%7CUnknown%7CTWFpbGZsb3d8eyJWIjoiMC4wLjAwMDAiLCJQIjoiV2luMzIiLCJBTiI6Ik1haWwiLCJXVCI6Mn0%3D%7C3000%7C%7C%7C&sdata=vJuj41vMtgehQHTPLGBG47e4T2d3LhX%2BYkxGPh89NMI%3D&reserved=0)).”

*4. In the discussion, could the authors please describe how they envision that CHD arises from deletion of Ift140 in multiple cell lineages.*

This concern was also raised by reviewer #1: comment 3. Discussion text revised to discuss this in detail. Excerpt of revised text from discussion can be found above in answer to reviewer #1.

**Reviewer #3:**

Major points

*1. Need a summary of the alleles and drivers used in this study- a schematic of where the null allele and where the point mutant 220 lie. Is this different than the Cauli allele mentioned line 111? Someway to schematically represent a lot of really complicated crosses and alleles. KO line- lines 136-137: truncated protein, but you show protein null. Consider aligning the narrative with lines 173. Similarly some figures, most of the episcopic confocal microscopy 3D or 2D are labelled p/d (flox/null1), and should be CAG-Cre? Null1 what about null2- do we need the number? It is confusing for readers. Nomenclature through all figures and text should be standardized- alleles should be in italics and title case for mouse, not all caps (i.e. Figure 3,6,7,8,9). (Formatting and nomenclature throughtout this manuscript need careful attention- but these text boxes allow neither italics or superscript frustratingly)*

New S2 Table added to manuscript to list all Cre lines used, including promotor, tissue expression, embryonic day expressed, MGI symbol, and citation.

New S6 Figure was generated (schematic of *Ift140* alleles used). *Ift140^220^* is different to the Cauli mutant mentioned in introduction.

New S3 Table added to manuscript (Summary of mouse breeding used to generate embryos analysed in this study)

We appreciate the comment regarding the need to standardize nomenclature and have now done so throughtout as suggested by the reviewer.

*2. CRE excision variability, dynamics (protein pool turnover) and lack of CHD phenotype. This alluded to with the Foxa2mcm allele mosaic recombination and lack of heart phenotypes upon tamoxifen treatment. These experiments were not done with a reporter Z/AP or Z/EG mT/mG, and ciliation in target tissues was not looked at, is possible that cardiac phenotypes are masked by variability in recombination levels, there needs to be a caveat in the text.*

We believe it is unlikely that there is extensive mosaicism in Cre recombination accounting for the complete failure to observe any CHD phenotypes, especially given this same result was seen with two different Cre drivers. However, as we cannot formally exclude this possibility, we have revised the Discussion to offer this as another possibility as shown below.

Revised Discussion addressing this point:

"While the CRE lines used in this study are well established and extensively utilized, the possibility exists that cardiac phenotypes were masked by variability in recombination levels and incomplete knockdown of ciliation in target tissues."

*3. Human ciliopathies- be consistent and maybe consider OMIM terms for the skeletal dysplasias you mention frequently. Sometimes its SRTD sometimes SRP, Sensenbrenner sometimes craniosyntosis- helps non-experts keep up.*

Text updated and ciliopathies names made more consistent as kindly suggested. OMIM terms also inserted into text.

Minor points

*1. Nomenclature: Cre lines should be italicized throughout Tbx18-Cre or Wnt1-Cre (lines 43-44). Table 2 needs revising. Use the correct line nomenclature for clarity 'tamoxifen (Tmx)-inducible Cag-CreER' is this the one from the Macmahon lab, then it should be CAGGCre-ER (line 249). The FoxA2-Cre is the knock-in allele from the Moon lab, then it should be Foxa2^mcm^.*

New S2 Table added to manuscript to list all Cre lines used. Cag-CreER in all text updated for CAGGCre-ER as suggested. FoxA2-Cre updated in all text to MGI synonym Foxa2Cre-ER (https://www.informatics.jax.org/allele/MGI:3774420) as it was considered important that "Cre" was kept in line name for better clarity for reader.

*Is the Gli1lz the knock-in reporter from the Joyner lab and the knock-in Ptch1lacZ from the Scott lab? For example line 469 uses two different nomenclatures for the same allele. If you need to explain the allele and don't want to do so in the text, include it in the methods. There are alternates to all of these and it is important to be able to clearly identify which one was used here.*

Methods section updated to list exact *Gli1-LacZ* (Gli1tm2Alj/J ; Jackson Laboratory Strain #:008211) and *Ptch1-LacZ* (Ptch1tm1Mps/J ; Jackson Laboratory Strain #:003081) strains used.

*2. Nomenclature: reference to Hedgehog related processes in mammals needs attention. Mammalian proteins ALL CAPs, mouse genes Title case italics. Introduce as Hedgehog (Hh). i.e. GLI, Patched1 (Ptch1) gene/mRNA or PTCH1 for protein. (Lines 55-62).*

Text updated as suggested

*3. All antibodies should be all caps IFT88 (line 180), PAX6, OLIG2, NKX6.1 (line 211). Typo in OLIG2 (not OLIGO2). Figure 4C,E- use the antibody name, not structure labelled gamma tub not alpha.*

Text/Figure updated as suggested

*4. Figure S2- control sections are not age or level matched? Wild type is earlier or more caudal? Induction of ventral populations change with time. Figure 5B- should NKX6.1 not be excluded from the FP in controls, only V3 and MN? Put in text line 207 what developmental stage was looked at?*

Unfortunately, we can't confirm the axial level was matched for the images shown in this figure, and thus we removed Figure S2 and reference to this analysis from the manuscript.

*5. Line 285/286- Clarify 'embryos collected at E12.5-14.5 from Tmx treatment at E7.5 had more severe phenotypes than than those collected at E16.5?' Why would this be the case- surely phenotypes would be consistent across developmental ages if treated at the same time, unless embryos are dying earlier.*

Text updated to clarify reviewer comment. Excerpt of revised text from Results:

"Consistent with this, embryos collected at E12.5-14.5 from Tmx treatment at E7.5 had more severe range of phenotypes than those collected at E16.5, suggesting a subset of embryos were likely dying and resorbed before harvest at E16.5. "

*6. Units on all blots should be kDa, not kD.*

Figures updated as suggested

*7. Line 337 should be Gli3Xt-j and craniosyntosis phenotypes are common in many syndromic ciliopathies affecting IFT.*

Text fixed as suggested

*8. Line 359: Can you separate lung underdevelopment from lack of a diaphragm and liver within the thoracic cavity? This is not what causes narrowed ribs in most SRTD/JTD patients, where bone growth is cause. This statement likely needs revision.*

Text revised to include this possibility. Excerpt of revised text from discussion:

"Lung development appeared developmentally arrested and is associated with a very small chest reminiscent of the thoracic dystrophy seen in SRTD. This lung underdevelopment could be related to the lack of a diaphragm and the marked expansion of liver tissue into the thoracic cavity of these embryos, which may physically prevent normal lung growth and expansion."

*9. Line 382 requires a reference: 'IFT140 ciliopathy patients often are compound heterozygous, with one allele being null and a second allele with a missense mutation.'*

Missing references (PMID 27874174; 32007091) added to text

*10. Line 412 'precise role of Ift140 in hedgehog signaling may be context dependent.' COuldn't it also be to do partial reduction in IFT pools in your CRE deleted lines- hypomorphic versus null?*

Possibility added to text in discussion. Excerpt of revised text from discussion:

Barring possible issues using Cre deleted models (where IFT140 may have been hypomorphic instead of null), the precise role of Ift140 in hedgehog signaling may be context dependent, with OFT development mediated by a non-cell autonomous role of cilia in the integration of multiple cilia transduced cell signaling pathways across different cell lineages.

*11. Line 425 Omphalocoele is not neural crest defect people think of.  It may be useful to expand a little that some skeletal muscles have neural crest contribution where long range migration of muscle precursors is required for lateral and body wall muscles needed for body wall closure. Please check this section.*

We thank the reviewer for pointing out the neural crest original of skeletal muscle, and thus its likely role in the body wall closure defect. We have now revised the Discussion to elaborate on this important point, with an excerpt of this revised text is shown below:

The observation that both the craniofacial and body wall closure defects were replicated by *Tbx18-Cre* and *Wnt1-Cre* would suggest these phenotypes arise from the perturbation of the cranial and trunk neural crest respectively and may be cell autonomous. We note trunk neural crest cells migrating through the rostral scleratome compartment targeted by the Tbx18-Cre has been shown to give rise to skeletal muscle, with those migrating to form the ventral dermomyotome differentiating into hypaxial skeletal muscle of the diaphragm, abdominal wall and limb [58]. Hence, it is likely that the loss of such trunk neural crest derived hypaxial musculature underlies the body wall closure defect observed with Tbx18-Cre deletion of *Ift140*. As *Tie2-Cre* also yielded omphalocele, this suggests blood vessels that are segmentally aligned with the sclerotome may also provide migratory cues to direct migration of neural crest cells to the ventral dermomyotome forming hypaxial muscles required for ventral body wall closure. As the rostral sclerotome will give rise to the ribs, whether trunk neural crest perturbation may contribute to thoracic dystrophy with shortened ribs warrants further investigation in future studies.

*12. Mouse breeding- lacking information on strain used.*

To clarify mouse breeding and strains used, Tables S2 and S3 were generated for manuscript

*13. Protein analysis- missing details of how protein lysates were made.*

Methods updated with missing details. Excerpt of revised text from methods:

"Embryos were dissected and dispersed in denaturing gel loading buffer (10 mM Tris pH 8.0, 16 mM dithiothreitol , 1 mM ethylenediaminetetraacetic acid, 5% sucrose, 1% sodium lauryl sulfate), passed through a 22 gauge needle to break up DNA, heated at 95 degrees C for 5 minutes, and separated by polyacrylamide gel electrophoresis. Proteins were transferred to Immobilon-P membrane (Merck Millipore Ltd.) and westerns preformed with Ift140 [23] and γ-tubulin (GTU-88, Sigma) antibodies. Western blots were developed by chemiluminescence (Super Signal West Dura, Pierce Thermo) and imaged using a LAS-3000 imaging system (Fujifilm) or film. Anti-MmIFT140 was made by expressing the last 356 residues of the mouse protein in bacteria as a maltose binding protein fusion and injecting into rabbits. Antibodies were affinity-purified against the same fragment expressed as a glutathione S-transferase fusion [23]."

*14. Line 820- instead of using the clone, use the antigen- acetylated tubulin?*

text fixed as kindly suggested

*15. Figure s3 legend- which Cre was used? What is loaded on A- lysates of which stage (representative to quantification below)? This could be clearer.*

Information included in S3 legend as suggested

*16. Figure S6- bottom label should reference Figure S3, Figure 4B- is gamma tubulin the loading control for the right blot- running of bands looks different and membrane looks cut? Labels on all say gamma tubulin and in legend but on blots labelled as alpha tubulin? Not specified in Fig 4 legend. Needs attention.*

Figures updated as suggested

Edits 1-16

*1. CHD is plural- line 41. Surprisingly, CHD were not observed…*

*2. Line 48- Plural for ciliopathies- many are affected by CHD.*

*3. Line 82: italicize Ift140 - mutations are in genes.*

*4. Line 92: E13 is mid-gestation, not early.*

*5. Line 159: wildtype littermates.*

*6. Line 180: do you mean centrioles instead of centrosomes ?*

*7. Line 192: use micron symbol.*

*8. Line 247: Should read gain-of-function.*

*9. Line 263: Clarify: it reads 'embryonic lethality, necessitating earlier embryo collection at E11.5-E12.5'. Do you mean neonatal lethality or that mutant embryos were missing at birth? Clearer ways to say this.*

*10. Line 355: Clarify 'Both Ift140 mutants exhibited a similar wide array of SBD, even though IFT-A is generally considered less critical for ciliary assembly than IFT-B'. Cilia don't assemble properly if there is no IFT-A, as you have shown?*

*11. Line 444: should be short rib polydactyly (SRP).*

*12. Line 515: transcripts should be italics (see also Line 837)*

*13. Lines 768, 770: italicize Ift140220*

*14. Line 772: space between value and unit. (For all figure legends)*

*15. Line 774: italicize alleles in all figure legends*

*16. Line 895: craniofacial?*

All minor edits suggested by reviewer #3 were conducted as suggested.
